# Haptoglobin Regulates Macrophage/Microglia‐Induced Inflammation and Prevents Ischemic Brain Damage Via Binding to HMGB1

**DOI:** 10.1161/JAHA.121.024424

**Published:** 2022-03-04

**Authors:** Mayuka Morimoto, Takafumi Nakano, Saki Egashira, Keiichi Irie, Kiyoshi Matsuyama, Momoka Wada, Yoshihiko Nakamura, Yutaka Shigemori, Hiroyasu Ishikura, Yuta Yamashita, Kazuhide Hayakawa, Kazunori Sano, Kenichi Mishima

**Affiliations:** ^1^ Faculty of Pharmaceutical Sciences Department of Physiology and Pharmacology Fukuoka University Fukuoka Japan; ^2^ Department of Pharmacy Almeida Memorial Hospital Oita Japan; ^3^ Departments of Radiology and Neurology Neuroprotection Research Laboratory Massachusetts General Hospital and Harvard Medical School Charlestown MA; ^4^ Faculty of Engineering Fukuoka Institute of Technology Fukuoka Japan; ^5^ Department of Emergency and Critical Care Medicine Fukuoka University Hospital Fukuoka Japan; ^6^ Department of Sports Medicine Faculty of Sports and Health Science Fukuoka University Fukuoka Japan

**Keywords:** cerebral ischemia, haptoglobin, high‐mobility group box 1, macrophage/microglia polarization

## Abstract

**Background:**

HMGB1 (high‐mobility group box 1) is known to worsen the functional prognosis after cerebral ischemia. Hp (haptoglobin) binds and sequesters HMGB1. Furthermore, Hp‐HMGB1 complexes are rapidly cleared by scavenger receptors on macrophages/microglia and modulate polarization of macrophages/microglia toward the M2 phenotype. Therefore, Hp may prevent aggravation by HMGB1 after cerebral ischemia and promote tissue repair by M2 macrophages/microglia. The aim of this study was to investigate the effects of Hp on ischemic brain damage induced by a high systemic HMGB1 level in mice subjected to 4 hours of middle cerebral artery occlusion (MCAO).

**Methods and Results:**

One day after MCAO, Hp was administered intraperitoneally at a dose of 20 or 200 U/kg once daily for 7 days. Neurological scores, motor coordination, and plasma HMGB1 levels were measured 1, 3, and 7 days after MCAO. Expression of M1 and M2 macrophage/microglia markers, such as CD16/32 and CD206, were evaluated by immunostaining 7 days after MCAO. Treatment with Hp for 7 days improved the neurological score, motor coordination, and survival and prevented brain damage after MCAO. The systemic HMGB1 level increased 1 to 7 days after MCAO and was higher at 7 days than at day 1. Hp significantly decreased the systemic HMGB1 level and increased the M2 phenotype when compared with the M1 phenotype after MCAO.

**Conclusions:**

Hp improved functional outcomes, including survival, motor function, and brain damage by binding to HMGB1 and modulating the polarization of macrophages/microglia. Hp may be an effective option in the treatment of cerebral ischemia.

**Nonstandard Abbreviations and Acronyms**
HMGB1: high‐mobility group box 1
Hp: haptoglobin
MCAO: middle cerebral artery occlusion
QCM: quartz crystal microbalance

**Clinical Perspective**

**What Is New?**

- Treatment with Hp (haptoglobin) for 7 days improved functional outcomes, including neurological function, motor coordination, and survival, even when started 24 hours after the onset of cerebral ischemia via binding to HMGB1 (high‐mobility group box 1).
- Hp regulates the macrophage/microglia‐induced proinflammatory and anti‐inflammatory responses after cerebral ischemia.

**What Are the Clinical Implications?**

- HMGB1 is a therapeutic target for the neuroinflammatory stage after cerebral ischemia
- Hp may be a novel treatment option for patients in the neuroinflammatory stage after ischemic stroke.

Extracellular HMGB1 (high‐mobility group box 1) acts as a cytokine‐like mediator that links early acute neuronal death in the ischemic core and subsequent brain damage associated with inflammatory responses in the ischemic penumbra.^1^ A large amount of HMGB1 is released by necrotic cells and infiltrating macrophages after cerebral ischemia and triggers inflammatory processes, such as activation of macrophages/microglia.^1^, ^2^, ^3^, ^4^, ^5^ Moreover, high expression of systemic HMGB1 increases the classically activated macrophages/microglia (M1), which release proinflammatory cytokines such as tumor necrosis factor‐alpha (TNF‐α), leading to progression of ischemic brain damage and a worsening of the functional prognosis.^6^, ^7^ We have previously demonstrated attenuation of brain damage and decreased M1 macrophages/microglia when recombinant human soluble thrombomodulin, which can decrease systemic HMGB1 levels, is administered in mice subjected to middle cerebral artery occlusion (MCAO).^7^ Therefore, inhibition of elevation of HMGB1 and upregulation of M1 macrophages/microglia may represent a novel therapeutic target in the neuroinflammatory stage after cerebral ischemia.

Hp (haptoglobin) is a plasma glycoprotein that binds to free hemoglobin and protects against iron‐induced oxidative damage, inflammation, and cerebrovascular disease.^8^ In Japan, a commercially developed Hp preparation (haptoglobin I.V. 2000 units; JB, Japan Blood Products Organization, Tokyo, Japan) was approved for the treatment of hemoglobinemia and hemoglobinuria induced by hemolysis in 1985. Hp is composed of 2 or 3 α and β subunits linked by disulfide bonds and exists as 3 phenotypes: Hp1‐1, Hp2‐2, and Hp2‐1.^9^ The Hp β subunit has high affinity for HMGB1 and inhibits HMGB1‐induced secretion of TNF‐α from macrophages.^9^, ^10^ Hp‐HMGB1 complexes are rapidly cleared by the CD163 scavenger receptor on macrophages and modulate polarization of macrophages toward the M2 phenotype.^9^, ^10^ The M2 phenotype is described as an alternative activation state involved in resolution of inflammation, tissue remodeling, and repair via secretion of heme oxygenase‐1 and interleukin‐10 (IL‐10).^9^, ^11^ Therefore, Hp may not only prevent the toxic and proinflammatory actions of HMGB1 but also promote repair of tissue by M2‐macrophages/microglia in ischemic stroke.

The aims of this study were to (1) investigate the effects of Hp on ischemic brain damage caused by elevation of HMGB1 and (2) clarify the role of Hp in polarization of the macrophages/microglia associated with elevated HMGB1 by investigating expression of the M1 and M2 phenotypic markers and proinflammatory cytokine levels after treatment with Hp in mice subjected to 4 hours of MCAO. Overall, our findings suggest that Hp is a potential therapeutic option against brain damage in the neuroinflammatory stage after cerebral ischemia.

## Methods

All data that support the findings of this study are available from the corresponding author upon reasonable request.

### Animals

All procedures on animal care and use were approved by the Experimental Animal Care and Use Committee of Fukuoka University. A total of 194 male ddY mice (age, 6–8 weeks; weight 25–35 g; Japan SLC Inc., Shizuoka, Japan) were used in this study. Data were obtained from mice based on their recovery following a successful MCAO surgery and survival to day 1 after MCAO (MCAO, n=127; sham, n=17; Pre, n=8). The Pre mice were the same as the sham‐operated mice. A successful MCAO surgery was determined by approximately an 80% reduction in regional cerebral blood flow from the baseline before MCAO surgery. Mice were excluded from analysis if they failed to achieve this regional cerebral blood flow (n=22) or died in the first 24 hours after MCAO (n=20). From all mice subjected to MCAO successfully (n=127), 85 mice were used to evaluate the functional outcomes at days 1 and 7 after MCAO, 7‐day survival rate, and histological and protein assay of day 7 samples. The remaining 42 mice were used for the brain and plasma protein assay of day 1 and 3. The detailed study protocol is shown Figures S1 and S2.

### Focal Cerebral Ischemia

Focal cerebral ischemia was induced in male ddY mice as described previously.^7^ The mice were anesthetized with 4.0% isoflurane (Pfizer, Osaka, Japan) and maintained by 1.5% isoflurane in 70% N_2_O and 30% O_2_. After a midline neck incision, 8‐0 nylon monofilament (Echilon; Johnson & Johnson, Tokyo, Japan) coated with silicone resin (Provil novo; Heraeus, Tokyo, Japan) was introduced through a small incision in the common carotid artery and advanced to a position 9 mm distal from the carotid bifurcation for occlusion of the middle cerebral artery. Rectal temperature was maintained between 36.5°C and 37.5°C. Cerebral ischemia was determined to be adequate using a laser Doppler flowmeter (Advance Co., Tokyo, Japan) and by assessment of forelimb flexion after the mice recovered from anesthesia. Four hours after occlusion, the mice were re‐anesthetized, and reperfusion was established by withdrawal of the filament.

### Drug Preparation and Administration

Haptoglobin I.V. 2000 units (JB) was dissolved in saline and immediately injected intraperitoneally at a dose of 20 or 200 U/kg once daily for 7 days starting on day 1 after MCAO. Mice were randomized into a sham group, a vehicle‐treated control (saline) group, an Hp 20 U/kg group, and an Hp 200 U/kg group. All analyses were performed by investigators who were masked to group allocation.

### Functional Outcomes

The functional outcomes evaluated were neurological deficit and motor coordination. Neurological deficit was evaluated at 1 and 7 days after MCAO using a previously described neurological score.^7^ Briefly, the scores were divided into 6 categories: 0, normal motor function; 1, flexion of the torso and the contralateral forelimb on lifting the animal by the tail; 2, circling to the ipsilateral side but normal posture at rest; 3, circling to the ipsilateral side; 4, rolling to the ipsilateral side; and 5, leaning to the ipsilateral side at rest (no spontaneous motor activity). Motor coordination was measured using the Rota‐Rod test on 1 and 7 days after MCAO, as described elsewhere.^12^

### Analysis of Loss of Brain Tissue

We assessed loss of brain tissue as an indicator of brain damage 7 days after MCAO. Each murine brain was sectioned at 2‐mm intervals using a brain matrix. To quantify the degree of brain loss, we compared the area of the striatum in the ipsilateral hemisphere with that on the contralateral side in each sample using ImageJ software (National Institutes of Health, Bethesda, MD). Tissue loss volume (%)=(contralateral area ‐ ipsilateral area)/contralateral area × 100%.

### TUNEL Staining and Quantification

Mice were anesthetized and perfused with saline and 4% paraformaldehyde 7 days after MCAO. Apoptosis was analyzed using a TUNEL (terminal deoxynucleotidyl transferase‐mediated dUTP nick‐end labeling) assay kit (Promega, Tokyo, Japan) according to the manufacturer’s instructions and as described previously.^7^ We randomly selected and photographed 3 areas of the peri‐infarct region (upper, middle, and bottom of the striatum) under a microscope (BZ‐X710; Keyence, Osaka, Japan). Images were collected using imaging software (BZ‐X analysis application; Keyence). All images were processed using the ImageJ software analysis system in a masked manner for unbiased counting, as described in a previous report.^7^ Mean TUNEL‐positive stained cell counts were calculated for the 3 microscopic fields in the peri‐infarct regions of each section, and sections were analyzed for each brain. Data are shown as the mean number of cells per square millimeter.

### Systemic HMGB1 Assay

Blood samples were collected at 1, 3, and 7 days after MCAO. These mice were anesthetized by isoflurane and blood samples were collected (0.5–0.7 mL) from the inferior vena cava. Plasma was obtained after centrifugation at 1500 rpm for 10 minutes at 4°C. Plasma HMGB1 levels were measured by ELISA (Shino‐Test Corp., Kanagawa, Japan) according to the manufacturer’s instructions.

### Immunofluorescence Staining and Quantification

The mice were anesthetized and perfused with saline and 4% paraformaldehyde 7 days after MCAO. The brains were cut into 25‐μm sections using a vibrating blade microtome (Vibrating Microtome 7000smz‐2; Campden Instruments Ltd., Loughborough, UK), as described previously.^12^ The sections were incubated overnight at 4°C with rabbit polyclonal anti‐Iba1 (ionized calcium‐binding adapter molecule 1) primary antibody (1:1000; Abcam, Cambridge, MA) as a marker of activated macrophages/microglia, rat polyclonal anti‐CD16/32 primary antibody (1:200; BioLegend, San Diego, CA) as a marker of the M1 phenotype, and goat polyclonal anti‐MMR/CD206 primary antibody (1:200; BioLegend) as a marker of the M2 phenotype. Sections were then incubated with Alexa Fluor 488‐/594‐labeled secondary antibodies (1:1000; Invitrogen, Carlsbad, CA) for 2 hours. The coverslips were mounted using ProLong Diamond Antifade Mountant with DAPI (Invitrogen), and the slides were observed under the BZ‐X710 microscope. We randomly selected and photographed 3 areas of the peri‐infarct region (upper, middle, and bottom of the striatum) under the microscope. Images were collected using imaging software (BZ‐X analysis application; Keyence). All images were processed using ImageJ software in a masked manner for unbiased counting, as described elsewhere.^7^, ^12^ Mean Iba1 and CD16/32 or CD206 positively stained cell counts were calculated from the 3 microscopic fields in the peri‐infarct regions of each section. The sections were then analyzed for each brain. The results for CD16/32+ Iba1/Iba1‐positive cells and CD206+ Iba1/Iba1‐positive cells were calculated based on the number of cells counted.

### Assay of Brain Cytokines and Hp

We performed a quantitative analysis of TNF‐α, IL‐10, and Hp levels in the brain for the sham, vehicle, and Hp 200 U/kg groups. Tissue samples were collected from the cortex at 7 days after MCAO. The total protein concentration in each tissue lysate was determined using the protein assay Bradford reagent (Wako Pure Chemical Industries, Fukuoka, Japan), as described previously.^7^ Tissue protein (20 μg) was measured by ELISA (TNF‐α and IL‐10, R&D Systems, Minneapolis, MN; Hp, Life Diagnostics, West Chester, PA) according to the manufacturer’s instructions.

### Absorption of HMGB1

We used a quartz crystal microbalance (QCM) to detect interactions between Hp and HMGB1 in vitro, as described previously.^7^ The oscillation frequency response of the QCM to the mass loaded on the electrode surface follows Sauerbrey Equation (1):^13^

(1) $$\Delta F=‐\frac{2F_{0}^{2}}{\sqrt{\rho_{Q}\mu_{Q}}}\frac{\Delta m}{A}$$

where Δ*F* is the amount of change in frequency, *F*_0_ is the basic frequency, Δ*m* is the amount of change in mass, *ρ* *_Q_* and *μ* *_Q_* are the density and quartz shear modulus values, respectively, and *A* is the active area of the crystal. HMGB1 and Hp were diluted with 1 mM 4‐(2‐hydroxyethyl)‐1‐piperazineethanesulfonic acid. First, the base mass was measured after 100 μg/mL of HMGB1 (Sigma, St. Louis, MO) was adsorbed onto the crystal in the QCM chamber. Next, 50 μL of Hp (0.4 U/L) was added to the QCM chamber. If Hp binds to HMGB1 on the crystal, the mass increases, and the frequency decreases.

### Statistical Analysis

Samples were randomly collected and quantified in a masked manner. Sample sizes were predetermined by G*Power ver. 3.1.9.4 software with effect sizes based on our previous studies as well as pilot studies.^7^, ^12^ We used an ANOVA: fixed‐effect, omnibus, 1‐way analysis with input parameters of α=0.05 and power (1‐β)=0.8. All data except for neurological scores were presented as mean±SEM. Multiple comparisons were performed using the Tukey‒Kramer test after 1‐way ANOVA. Neurological scores were presented as median with range. Multiple comparisons of neurological scores were analyzed by the Kruskal‒Wallis test with Dunn multiple‐comparison test. All statistical analyses were performed using JMP version 12.0.1 (SAS Institute, Tokyo, Japan). A *P* value of <0.05 was considered statistically significant.

## Results

### Effects of Hp on Functional Outcomes and Survival After Cerebral Ischemia

**Figure 1:**
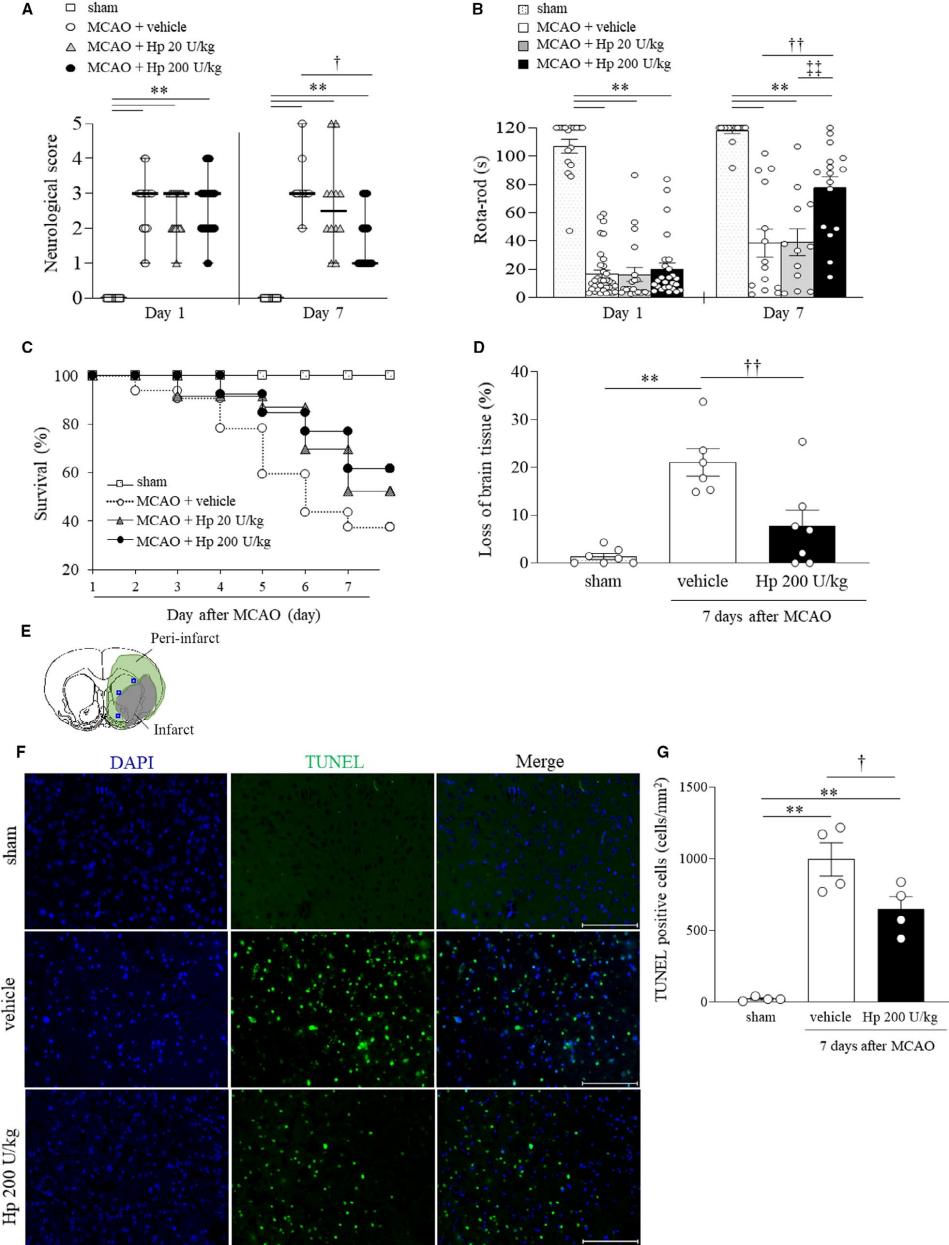
Hp (haptoglobin) prevents ischemic brain damage and improves the functional outcomes and survival after middle cerebral artery occlusion. Quantitative analysis of neurological impairment in the sham (n=17), vehicle, Hp 20 U/kg, and Hp 200 U/kg groups (day 1, n=36, 23 and 26; day 7, n=14, 12 and 16) (**A**). The data are presented as the median with range and analyzed by the Kruskal‒Wallis test followed by Dunn test. Quantitative analysis of performance on the Rota‐Rod test (**B**) and survival (**C**) in the sham (n=17), vehicle, Hp 20 U/kg, and Hp 200 U/kg groups (day 1, n=36, 23 and 26; day 7, n=14, 12 and 16). Quantitative analysis of loss of brain tissue (%) at 7 days after cerebral ischemia in the sham (n=7), vehicle (n=6), and Hp 200 U/kg groups (n=7) (**D**). The blue rectangles represent the areas observed to evaluate the fluorescent staining (upper, middle, and bottom of the striatum in the peri‐infarct region) (**E**). Representative image of fluorescent double staining of TUNEL (terminal deoxynucleotidyl transferase‐mediated dUTP nick‐end labeling) (green) and 4′,6‐diamidino‐2‐phenylindole (blue) at 7 days after cerebral ischemia (**F**). Quantification of TUNEL‐positive cells in the sham, vehicle, and Hp 200 U/kg groups (n=4 per group) (**G**). Scale bar, 100 μm. All values except for neurological scores are presented as the mean±SEM and analyzed by the 1‐way analysis of variance with Tukey test. ^**^ *P*<0.01 versus sham; ^†^ *P*<0.05, ^††^ *P*<0.01 versus vehicle; ^‡‡^ *P*<0.01 versus Hp 20 U/kg. DAPI, 4′,6‐diamidino‐2‐phenylindole; HP, haptoglobin; MCAO, middle cerebral artery occlusion; and TUNEL, terminal deoxynucleotidyl transferase‐mediated dUTP nick‐end labeling.

Mice subjected to MCAO showed significantly impaired neurological function in comparison with the sham group (*P*<0.0001). Treatment with Hp 200 U/kg for 7 days significantly improved the neurological scores when compared with the vehicle‐treated control group (*P*=0.0304; Figure 1A). One‐way ANOVA of the Rota‐Rod test indicated significant overall differences in effect between the 4 groups at 7 days (*F*_3,55_=23.6566, *P*<0.0001; Figure 1B). Treatment with Hp 200 U/kg for 7 days significantly improved performance on the Rota‐Rod test (*P*=0.0073; Figure 1B) when compared with the vehicle‐treated control group. Moreover, mice subjected to MCAO had a 7‐day survival rate of 38.9%. There was no significant difference in the survival rate at 7 days between the vehicle‐treated and Hp‐treated groups. However, Hp at a dose of 200 U/kg increased the survival rate by 22.6% (Figure 1C). Based on these results, we determined that 200 U/kg is the minimal effective dose of Hp based on the functional outcomes and survival after MCAO. Next, we investigated the histological and biological effects of Hp 200 U/kg.

### Effects of Hp on Severity of Brain Damage After Cerebral Ischemia

One‐way ANOVA of loss of brain tissue (*F*_2,17_=14.6712, *P*=0.0002) showed significant differences in overall effect between the 3 groups at 7 days (Figure 1D). Loss of brain tissue was significantly greater in the mice subjected to MCAO than in the sham group (*P*=0.0002) and was significantly improved in the mice treated with Hp 200 U/kg for 7 days (*P*=0.0056) when compared with the vehicle‐treated group. One‐way ANOVA of the number of TUNEL‐positive cells in the peri‐infarct region showed a significant difference in overall effect between the 3 groups (*F*_2,9_=34.6725, *P*<0.0001; Figure 1E and 1F). The number of TUNEL‐positive cells was significantly higher in mice subjected to MCAO than in the sham group (*P*<0.0001) and significantly lower in mice treated with Hp 200 U/kg than in the vehicle‐treated group (*P*=0.0204).

### Effects of Hp on Systemic HMGB1 Levels After Cerebral Ischemia

**Figure 2:**
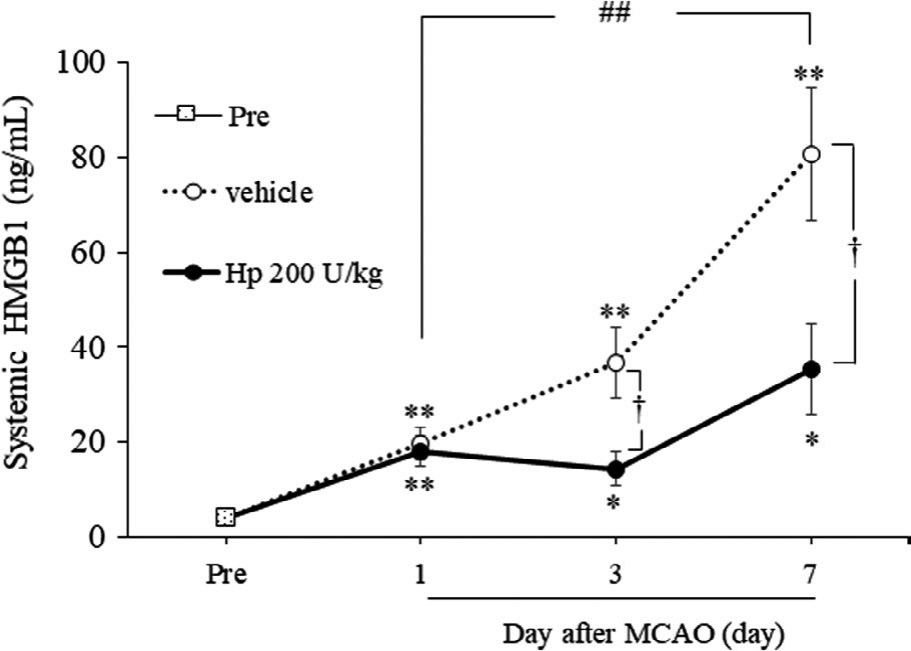
Treatment with Hp (haptoglobin) inhibits systemic elevation of HMGB1 (high‐mobility group box 1) after middle cerebral artery occlusion. Quantitative analysis of HMGB1 levels in the Pre (n=8), vehicle, and Hp 200 U/kg groups (day 1 and 3, n=10 per group; day 7, n=10 and 9 per group, respectively). HMGB1 indicates high‐mobility group box 1; Hp, haptoglobin; MCAO, middle cerebral artery occlusion. ^*^ *P*<0.05, ^**^ *P*<0.01 versus Pre; ^†^ *P*<0.05 vs vehicle; ^##^ *P*<0.01 vs vehicle on day 1 (Tukey test).

One‐way ANOVA of systemic HMGB1 levels on days 1 (*F*_2,25_=7.7105, *P*=0.0025), 3 (*F*_2,25_=10.0540, *P*=0.0006), and 7 (*F*_2,24_=13.4774, *P*=0.0001) after MCAO showed a significant difference in overall effect between the 3 groups (Figure 2). Mice subjected to MCAO showed significantly increased systemic HMGB1 levels on days 1 (*P*=0.0035), 3 (*P*=0.0006), and 7 (*P*<0.0001) after MCAO compared with the Pre group. We confirmed that there was no difference in systemic HMGB1 levels between the Pre group and the sham group. Moreover, systemic HMGB1 levels were higher on day 7 than on day 1 (*P*<0.0001) in mice subjected to MCAO. Systemic HMGB1 levels were significantly lower in the group treated with Hp 200 U/kg than in the vehicle‐treated group at 3 (*P*=0.0125) and 7 (*P*=0.0125) days after MCAO.

### Effects of Hp on Expression of Damaging Factors: M1 Macrophages/Microglia and Brain TNF‐α Levels at 7 days After Cerebral Ischemia

**Figure 3:**
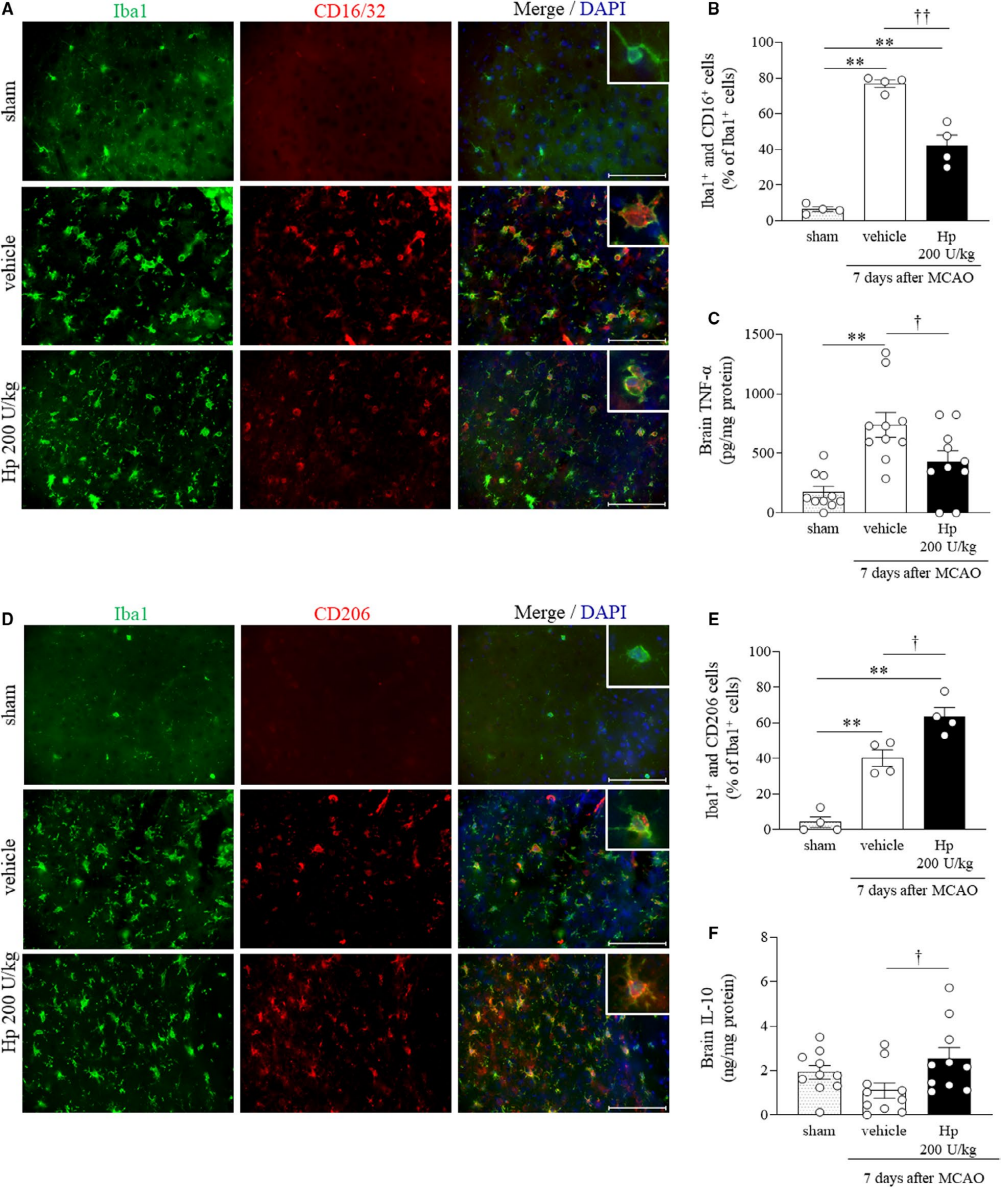
Treatment with Hp (haptoglobin) decreases the number of Iba1‐positive and CD16/32‐positive cells and expression of tumor necrosis factor‐alpha, and increases the number of Iba1‐positive and CD206‐positive cells and expression of interleukin‐10 in the brain 7 days after middle cerebral artery occlusion. Representative image showing the fluorescent double staining of Iba1 (green) and CD16/32 (red) 7 days after cerebral ischemia (**A**). The merged images depict CD16/32‐positive macrophages/microglia (orange). Quantification of cells double‐labeled with CD16/32‐and Iba1/Iba1‐positive cells in the sham, vehicle, and Hp 200 U/kg groups (n=4 per group) (**B**). Quantitative analysis of brain tumor necrosis factor‐alpha‐α levels in the sham, vehicle, and Hp 200 U/kg groups (n=10 per group) (**C**). Representative image of fluorescent double staining of Iba1 (green) and CD206 (red) 7 days after cerebral ischemia (**D**). Merged images depict CD206‐positive macrophages/microglia (orange). Quantification of cells double‐labeled with CD206 and Iba1/Iba1‐positive cells in the sham, vehicle, and Hp 200 U/kg groups (n=4 per group) (**E**). Quantitative analysis of brain IL‐10 levels in the sham, vehicle, and Hp 200 U/kg groups (n=10 per group) (**F**). Scale bar, 100 μm. DAPI indicates 4′,6‐diamidino‐2‐phenylindole; HP, haptoglobin; Iba1, ionized calcium‐binding adapter molecule 1; IL‐10, interleukin‐10; MCAO, middle cerebral artery occlusion; and TNF‐α, tumor necrosis factor‐α. ^**^ *P*<0.01 vs sham; ^†^ *P*<0.05, ^††^ *P*<0.01 vs vehicle (Tukey test).

One‐way ANOVA of CD16/32 immunoreactivity in Iba1‐positive cells in the peri‐infarct region at 7 days after MCAO revealed significant differences in overall effect between the 3 groups (*F*_2,9_=112.4383, *P*<0.0001; Figure 3A and 3B). CD16/32 immunoreactivity in Iba1‐positive cells was significantly greater in mice subjected to MCAO than in the sham group (*P*<0.0001) and significantly lower in mice treated with Hp 200 U/kg than in the vehicle‐treated group (*P*<0.0001). Moreover, 1‐way ANOVA of brain TNF‐α levels at 7 days after MCAO showed a significant difference in overall effect between the 3 groups (*F*_2,27_=11.0824, *P*=0.0003; Figure 3C). TNF‐α levels were significantly higher in mice subjected to MCAO than in the sham group (*P*=0.0002) and significantly lower in mice treated with Hp 200 U/kg than in the vehicle‐treated group (*P*=0.0420).

### Effects of Hp on Expression of Restorative Factors: M2 Macrophages/Microglia and Brain IL‐10 Levels at 7 Days After Cerebral Ischemia

One‐way ANOVA of CD206 immunoreactivity in Iba1‐positive cells in the peri‐infarct region at 7 days after MCAO revealed significant differences in overall effect between the 3 groups (*F*_2,9_=47.0453, *P*<0.0001; Figure 3D and 3E). CD206 immunoreactivity in Iba1‐positive cells was significantly higher in mice subjected to MCAO than in both the sham group (*P*=0.0006) and the vehicle‐treated group (*P*=0.0111). Moreover, 1‐way ANOVA of brain IL‐10 levels at day 7 after MCAO showed a significant difference in overall effect between the 3 groups (*F*_2,27_=3.4362, *P*=0.0468; Figure 3F). There was no significant difference in the brain IL‐10 level between mice subjected to MCAO and the sham group (*P*=0.3150). However, the IL‐10 level was significantly higher in the group treated with Hp 200 U/kg than in the vehicle‐treated group (*P*=0.0373).

### Change in Hp Levels in the Brain After Administration of Hp Following Cerebral Ischemia

**Figure 4:**
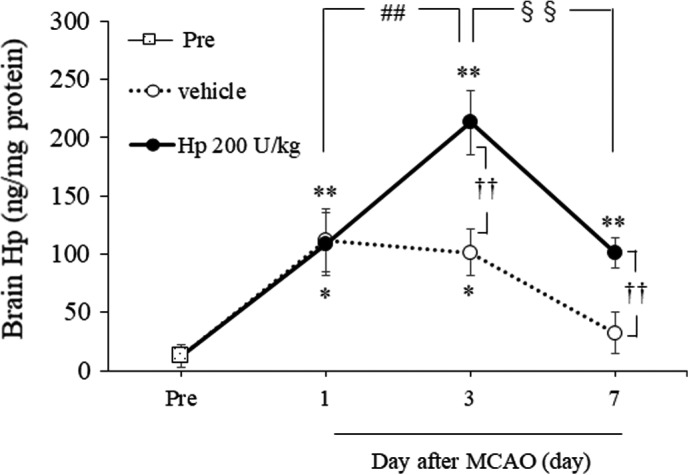
Increase in brain Hp (haptoglobin) levels after administration of Hp following cerebral ischemia Quantitative analysis of brain Hp levels in the Pre (n=8), vehicle, and Hp 200 U/kg groups (all days, n=8 per group). Hp indicates haptoglobin; and MCAO, middle cerebral artery occlusion. ^*^ *P*<0.05, ^**^ *P*<0.01 vs Pre; ^††^ *P*<0.01 vs vehicle; ^##^ *P*<0.01 vs Hp 200 U/kg on day 1; ^§§^ *P*<0.01 vs Hp 200 U/kg on day 3 (Tukey test).

One‐way ANOVA of Hp levels in the brain on days 1 (*F*_2,21_=6.6201, *P*=0.0059), 3 (*F*_2,21_=23.9495, *P*<0.0001), and 7 (*F*_2,21_=11.1767, *P*=0.0005) after MCAO revealed a significant difference in overall effect between the 3 groups (Figure 4). Brain Hp levels were significantly higher in mice subjected to MCAO than in the Pre group from day 1 (*P*=0.0091) to day 3 (*P*<0.0001) and decreased thereafter until day 7 (versus Pre group, *P*=0.5745). We confirmed that there was no difference in brain Hp levels between the Pre group and the sham group. Brain Hp levels were also significantly higher on days 3 (*P*=0.0026) and 7 (*P*=0.0059) in the group treated with Hp 200 U/kg than in the vehicle‐treated group.

### Effects of Hp on Absorption of HMGB1 In Vitro

**Figure 5:**
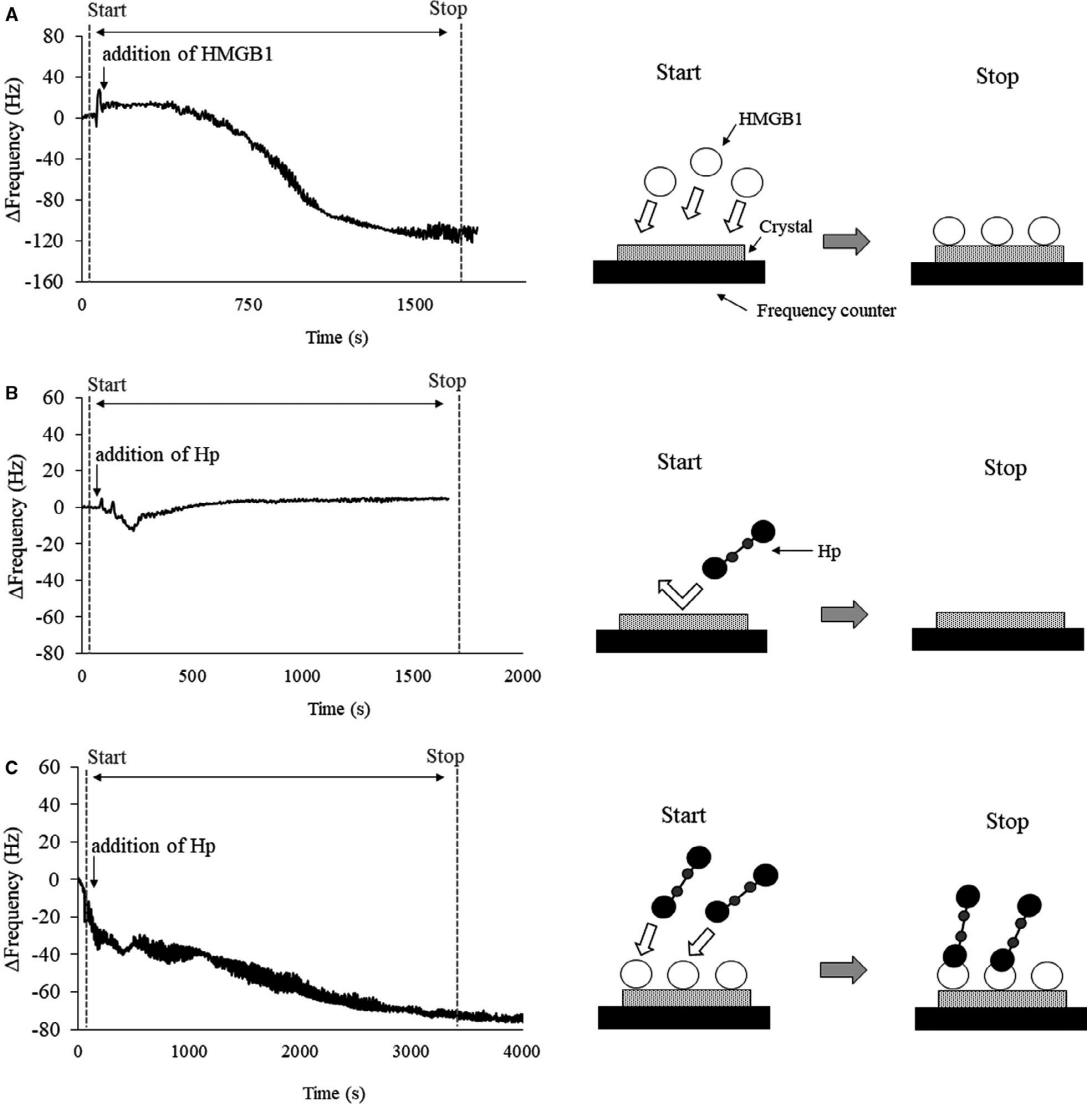
Hp (haptoglobin) binds rapidly to HMGB1 (high‐mobility group box 1). A quartz crystal microbalance was used to detect interactions between HMGB1 and Hp in vitro. **A through C,** are imaging illustrations. We confirmed the reproducibility of the results in triplicate. HMGB1 indicates high‐mobility group box 1; and Hp, haptoglobin.

First, we confirmed that HMGB1 alone was adsorbed on the crystal in the QCM chamber while Hp alone was not (Figure 5A and 5B). We considered this finding to reflect the hydrophobicity of Hp. Next, the base frequency was determined after HMGB1 was adsorbed onto the crystal in the QCM chamber. Δ*F* decreased rapidly after addition of Hp into the QCM chamber (Figure 5C); that is, Hp bound to HMGB1 on the crystal, thereby increasing the mass. Moreover, Hp bound rapidly to HMGB1. We confirmed the reproducibility of these results in triplicate.

## Discussion

This study evaluated the effects of Hp on the neuroinflammation and brain damage induced by HMGB1 after cerebral ischemia. Treatment with Hp for 7 days improved functional outcomes, including neurological function, motor coordination, and survival, even when started 24 hours after onset of cerebral ischemia. In this study, we identified that the mechanism via which Hp exerts its beneficial effect includes rapid binding to HMGB1, which prevents systemic elevation of HMGB1 after cerebral ischemia, decreases M1 macrophages/microglia, and suppresses brain damage. At the same time, the Hp‐HMGB1 complex could increase the M2 macrophages/microglia and expression of IL‐10 in the brain, also resulting in less brain damage. Our findings indicate that Hp not only prevents brain damage but also repairs tissue injury after cerebral ischemia. This is the first report to clarify the mechanism via which Hp protects against cerebral ischemia by inhibiting systemic elevation of HMGB1. Our observations suggest that Hp may be a novel treatment option for patients in the neuroinflammatory stage after ischemic stroke.

The gradual increase in systemic HMGB1 levels after the onset of cerebral ischemia occurs because of the release of HMGB1 from necrotic or damaged cells or from activated macrophages/monocytes.^1^ The released HMGB1 stimulates the receptors for advanced glycation end products and Toll‐like receptors expressed on neurons, mononuclear phagocytes, and glial cells, and promotes production of proinflammatory cytokines.^14^, ^15^, ^16^, ^17^, ^18^ Therefore, systemic HMGB1 is a potent proinflammatory mediator of cerebral ischemia. In our previous study, systemic HMGB1 levels increased between days 1 and 7 after MCAO and were higher on day 7 than on day 1. At the same time, the survival rate decreased from day 1 to day 7 after MCAO, and brain damage and M1 macrophages/microglia were increased.^5^, ^7^ Those findings indicated that systemic HMGB1 is an important determinant of the prognosis of cerebral infarction. Therefore, in this study, we started the administration of Hp 24 hours after cerebral ischemia when systemic HMGB1 levels began to increase and examined the protective effects of Hp in the brain. We found that treatment with Hp significantly improved the neurological deficit and motor dysfunction after cerebral ischemia by inhibiting systemic elevation of HMGB1, indicating that HMGB1 is a therapeutic target for the neuroinflammatory stage after cerebral ischemia and that Hp ameliorates ischemic brain damage by inhibiting systemic elevation of HMGB1.

We also demonstrated that treatment with Hp regulates macrophage/microglia‐induced inflammation after MCAO. High systemic HMGB1 levels are known to be responsible for activation of macrophages/microglia after cerebral ischemia.^19^, ^20^ Thus, Hp is considered to reduce systemic HMGB1 levels by forming a complex with HMGB1 and controlling the activation of macrophages/microglia. Activated macrophages/microglia include both an M1 type, which aggravates brain damage, and an M2 type, which repairs brain damage.^6^, ^21^, ^22^ M1 macrophages/microglia release inflammatory cytokines, including TNF‐α and IL‐6, whereas M2 macrophages/microglia release repairing cytokines, such as IL‐10. Our study showed that treatment with Hp significantly decreased the number of Iba1‐positive and CD16/32‐positive cells, markers of the M1 phenotype, and increased the number of Iba1‐positive and CD206‐positive cells, markers of the M2 phenotype, in comparison with vehicle‐treated mice. Therefore, Hp suppressed the increase in systemic HMGB1 that triggered the appearance of M1 macrophages/microglia, and it is probable that Hp lead to a decrease in these macrophages/microglia. In turn, Hp was able to reduce the expression level of TNF‐α and suppress the exacerbation of brain damage.

Hp is thought to protect the brain by suppressing cytotoxicity caused by HMGB1 and damage caused by M1 macrophages/microglia associated with elevated HMGB1. Furthermore, expression of CD206 in the brain was significantly increased by administration of Hp, along with the expression of IL‐10. The Hp‐HMGB1 complex has been reported to increase the number of M2 macrophages,^9^, ^10^ and it is suggested that Hp achieves this via binding to HMGB1 and enhancing their repair function in cerebral infarction. Hence, Hp may regulate the macrophage/microglia‐induced proinflammatory and anti‐inflammatory responses after cerebral ischemia. Both the reversal of inflammatory activity because of the action of the Hp‐HMGB1 complex and the reduction of M1 macrophages/microglia are thought to cause the relative increase in M2 macrophages/microglia. Our findings indicate that Hp not only inhibits injurious factors but may also increase levels of repair factors. We propose that Hp has the potential to become a therapeutic agent with a mechanism different from that of the conventional treatments presently available for cerebral infarction.

We focused on systemic elevation of HMGB1 because it reflected the severity of functional outcomes and the increase of M1 macrophages/microglia after cerebral ischemia.^5^, ^7^ Meanwhile, we have confirmed that the level of brain HMGB1 after cerebral ischemia also increases more slowly than the plasma HMGB1 level.^5^, ^7^ Thus, if Hp would be transferred into the brain, it is expected that it may also work to inhibit the function of brain HMGB1. Hp, a plasma protein, is produced primarily in the liver and the molecular weight of Hp varies according to the phenotype, but can be as large as 100–900 kDa; therefore, it might not normally pass through the blood‐brain barrier (BBB).^23^, ^24^, ^25^, ^26^ However, we have previously confirmed BBB leakage by immunoglobulin G and Evans blue extravasation in our ischemic mouse model.^7^ Similarly, it is possible that Hp could be transferred into the brain because of breakdown of the BBB after cerebral ischemia. Therefore, we examined the change in Hp levels in the brain after cerebral ischemia and found that Hp levels in the murine brain on days 1 and 3 after MCAO were significantly higher than those in the sham (Pre) group. These results indicate that the collapse of the BBB caused by MCAO resulted in movement of endogenous Hp that was circulating in the blood into the brain, resulting in a transient increase. However, the level of brain Hp was decreased 7 days after MCAO because of consumption of endogenous Hp because of systemic elevation of HMGB1 and decrease in BBB leakage over time. Activated microglia can be detected after as little as 20 minutes of reperfusion following cerebral ischemia, but immunohistochemical staining for these cells is pronounced after 24 hours, reaching maximal levels after 4 to 7 days.^5^, ^7^, ^27^ Thus, we consider that a high level of brain Hp needs to be maintained until at least 4 to 7 days after cerebral ischemia to affect the activated microglia. In this study, the level of brain Hp was significantly higher in the group treated with Hp than in vehicle‐treated mice at 3 and 7 days after MCAO, which indicates that exogenously administered Hp is transferred into the brain after the onset of cerebral ischemia. We revealed that Hp may act to protect the brain by regulating macrophage/microglia‐induced inflammation, not only systemically but also in the brain.

Nevertheless, there are a few caveats that should be addressed in future studies. First and foremost, we tested the effect of Hp in only young male mice. As stroke is an age‐associated disease,^28^ further studies using aged mice should be conducted to evaluate the beneficial action mediated by treatment with Hp in aging pathology. Moreover, sex differences may impact stroke outcome and therapeutic response, wherein women may have worse outcomes, increased disability, and decreased quality of life.^29^ Future studies should also include a female cohort to generalize our findings. Finally, demonstrating the presence of Hp‐HMGB1 complexes in the brain would greatly strengthen the conclusion that Hp improves the neuroinflammation by binding to HMGB1 rather than via some other functional property of Hp. Yang et al., reported that neither Hp alone nor HMGB1 alone increase the expression of IL‐10 in macrophages.^9^ They also showed that Hp needs to bind to HMGB1 to increase the expression of IL‐10 in macrophages. Our results showed that treatment with Hp significantly increased IL‐10 levels in the brain compared with that in the vehicle‐treated control group. The increased brain IL‐10 levels support that treatment with Hp may increase Hp‐HMGB1 complexes in the brain. Future study will be warranted to assess the brain level of Hp‐HMGB1 complex and the rigorous validation.

## Conclusions

The results of this study indicate that treatment with Hp improves functional outcomes, including survival, motor dysfunction, and brain damage, by binding to HMGB1 and modulating the polarization of activated macrophages/microglia. Hp may have the potential to become a therapeutic agent with a mechanism different from that of the conventional treatments presently available for cerebral infarction.

## Sources of Funding

This work was supported by funding from Fukuoka University (grant number 207107) and Japan Society for the Promotion of Science KAKENHI (grant number 20K07215).

## Disclosures

None.

## Supporting information

Figures S1–S2
