# Supplementary material for: Haptoglobin Regulates Macrophage/Microglia‐Induced Inflammation and Prevents Ischemic Brain Damage Via Binding to HMGB1
Source: J Am Heart Assoc. 2022 Mar 4;11(6):e024424. doi: 10.1161/JAHA.121.024424 (PMC9075294; doi:10.1161/JAHA.121.024424)
Supplement: Supplementary file 1 — Figures S1–S2 [file JAH3-11-e024424-s001.pdf]

SUPPLEMENTAL MATERIAL

Figure S1. Study protocol.

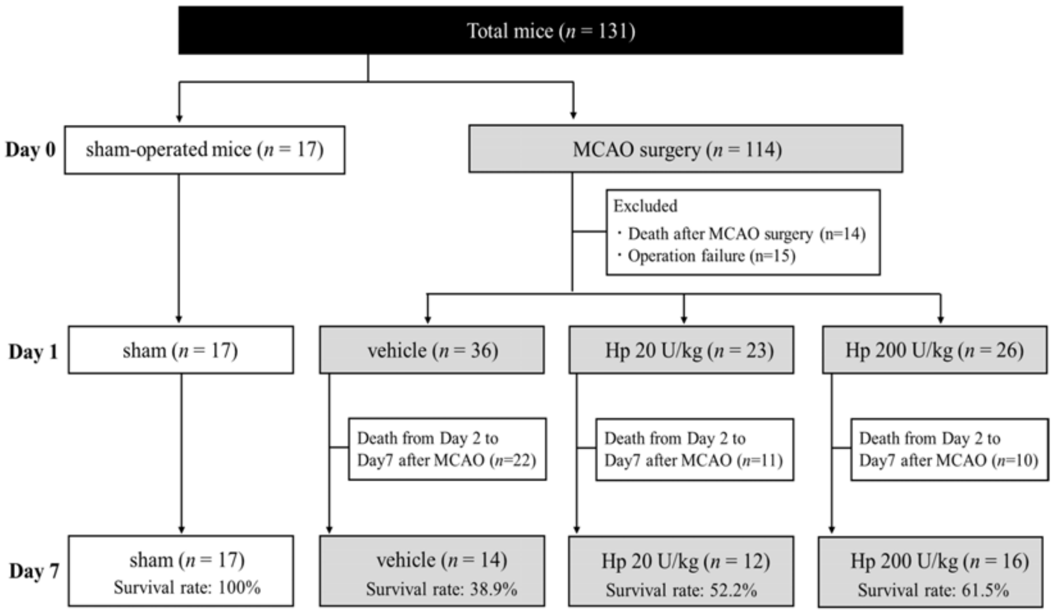

We evaluated the functional outcomes, 7-day survival rate and collected day 7 samples from this cohort. Hp indicates haptoglobin; MCAO, middle cerebral artery occlusion.

Figure S2. **Study protocol of collecting days 1 and 3 samples**

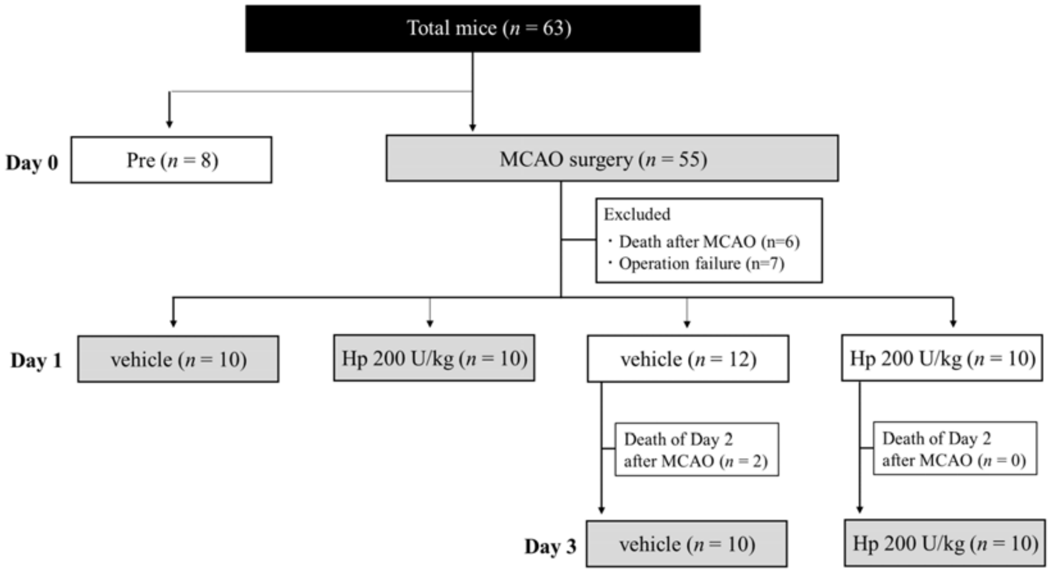

Hp indicates haptoglobin; MCAO, middle cerebral artery occlusion.
